# Supplementary material for: Negative Correlation between Lipid Content and Antibiotic Activity in Streptomyces: General Rule and Exceptions
Source: Antibiotics (Basel). 2020 May 26;9(6):280. doi: 10.3390/antibiotics9060280 (PMC7344866; doi:10.3390/antibiotics9060280)

Figure S1 : LC/Corona-CAD results of total lipid content in *Streptomcyces antibioticus* DSM 41481 and *Streptomyces sp*. DSM 40868 grown for 72h on solid R2YE glucose medium limited in phosphate. TAG stands for TriAcylGlycerol, DAG for DiAcylGlycerol, FA for Fatty Acids, PE for PhosphatidylEthanolamine, PA for Phosphatidic Acid, PI for Phosphatidyl Inositol, PIMs for PhosphatidylInositol Mannosides and CL for CardioLipid.

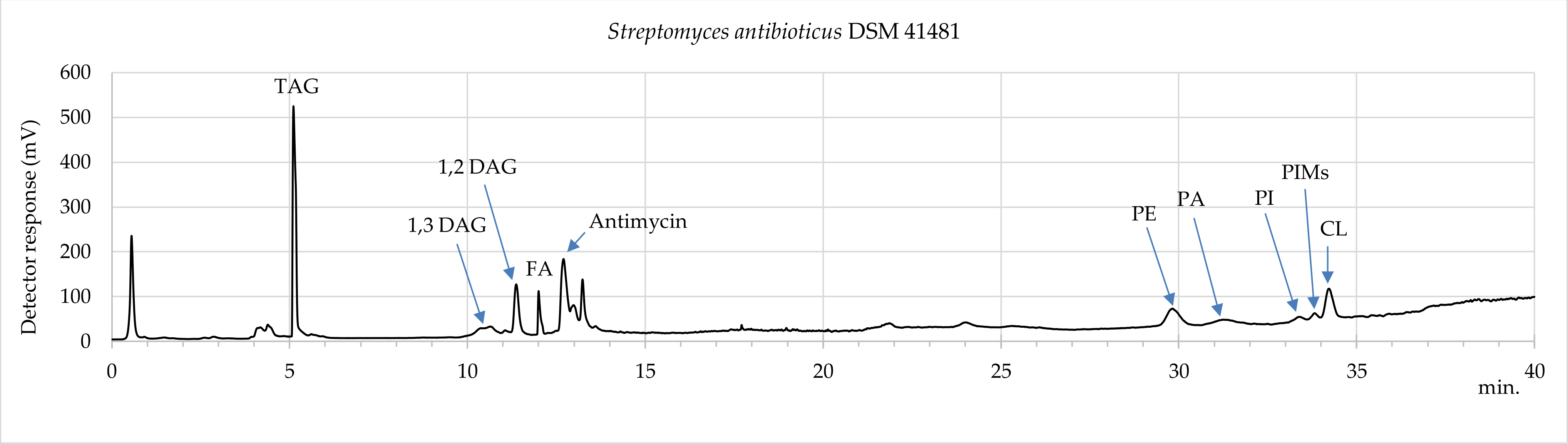

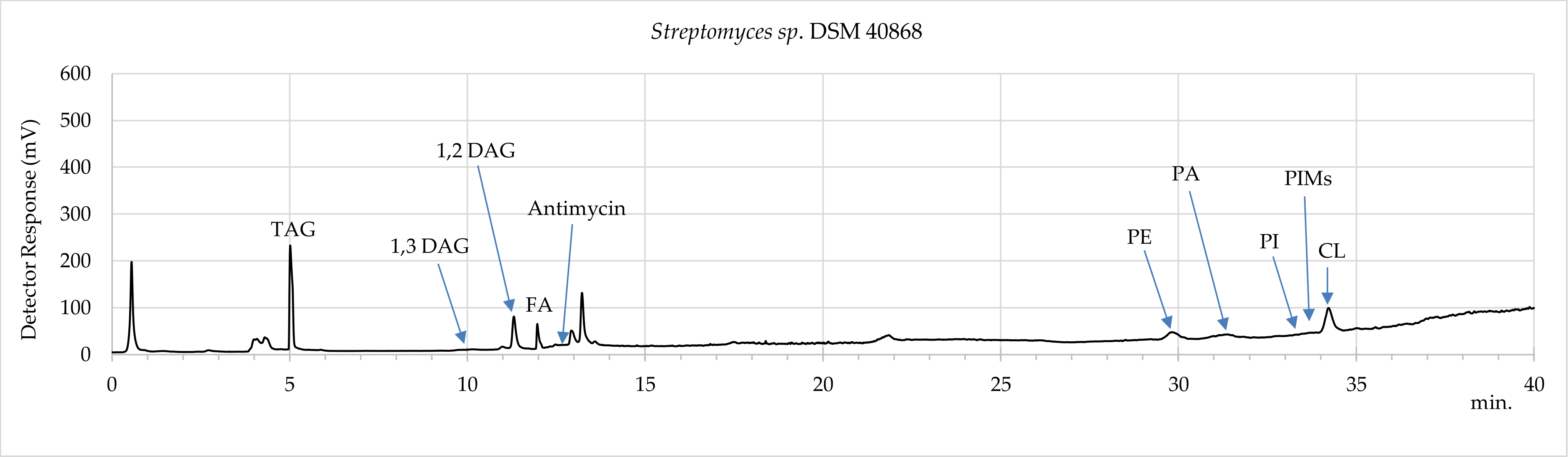

Figure S2 : Anti-Smash analysis of the genome of *Streptomyces antibioticus* DSM 41481 (8,473,575 bp).

Region	Type	From	To	Most similar known cluster		Similarity
Region 1	bacteriocin	216,990	226,748			
Region 2	lanthipeptide, terpene	327,516	361,829	Isorenieratene	terpene	100%
Region 3	terpene, melanin	686,666	707,133	Melanin	other	71%
Region 4	NRPS, T1PKS	731,747	800,687	Foxicins A–D	nrps-t1pks	12%
Region 5	other, CDPS, NRPS	1,217,807	1,292,777	Diisonitrile antibiotic SF2768	NRPS	50%
Region 6	terpene, NRPS-like, T1PKS, T2PKS	1,320,700	1,451,170	Spore pigment	t2pks	83%
Region 7	NRPS-like	1,943,835	1,985,314	Octacosamicin	nrps-t1pks	12%
Region 8	ectoine	2,173,921	2,184,325	Ectoine	other	100%
Region 9	melanin	3,218,444	3,228,956	Istamycin	saccharide	5%
Region 10	siderophore	3,311,605	3,322,761	Desferrioxamine	other	83%
Region 11	T2PKS	3,825,239	3,897,733	Fredericamycin	t2pks	81%
Region 12	furan, butyrolactone	4,452,480	4,476,188	Methylenomycin	other	14%
Region 13	terpene	5,656,885	5,677,058	Albaflavenone	terpene	100%
Region 14	NRPS-like	5,732,586	5,775,707	Chloramphenicol	NRPS	17%
Region 15	siderophore	6,330,896	6,342,710			
Region 16	bacteriocin	6,531,965	6,542,189			
Region 17	terpene, butyrolactone	6,571,015	6,592,442	Gamma-butyrolactone	other	100%
Region 18	siderophore	6,730,014	6,743,257	Grincamycin	t2pks-saccharide	5%
Region 19	NRPS, T1PKS	6,832,866	6,889,853	Antimycin	nrps-t1pks	100%
Region 20	indole	6,981,746	7,002,897	Terfestatin	other	23%
Region 21	terpene	7,194,476	7,221,197	Hopene	terpene	92%
Region 22	T1PKS, hglE-KS	7,247,598	7,298,972	Divergolide	t1pks	13%
Region 23	bacteriocin	7,520,677	7,530,892	Informatipeptin	lanthipeptide	42%
Region 24	NRPS	7,678,965	7,736,702	Scabichelin	NRPS	80%
Region 25	NRPS-like	7,929,006	7,970,314			
Region 26	other, NRPS	8,157,649	8,227,375	Actinomycin	NRPS	89%

Figure S3 : Anti-smash analysis of the genome of *Streptomyces sp.* DSM 40868 (registered in gene bank as *Streptomyces antibioticus* DSM 40868 or ATCC 11891) (9,195,693 bp).

Region	Type	From	To	Most similar known cluster		Similarity
Region 1	NRPS	54,676	133,284	Azicemicin	t2pks	13%
Region 2	lanthipeptide	142,326	165,105			
Region 3	NRPS	280,799	326,337	Paenibactin	NRPS	83%
Region 4	betalactone , NRPS-like , nucleoside	370,187	417,072	Nikkomycin	other	100%
Region 5	terpene	417,600	440,735	Carotenoid	terpene	63%
Region 6	lassopeptide , NRPS	546,976	606,763	Achromosin	other	100%
Region 7	lassopeptide	623,914	646,589	SSV-2083	lanthipeptide	37%
Region 8	butyrolactone	647,972	657,972			
Region 9	lanthipeptide , phenazine	684,123	720,741	Primycin	t1pks	8%
Region 10	T1PKS	950,214	1,062,317	Sceliphrolactam	t1pks	92%
Region 11	melanin	1,207,953	1,217,563	Melanin	other	57%
Region 12	T3PKS	1,645,922	1,685,386	Herboxidiene	t1pks+t3pks	6%
Region 13	terpene	1,721,550	1,740,878	4-Z-annimycin	t1pks	22%
Region 14	ectoine	2,346,568	2,355,857	Ectoine	other	100%
Region 15	terpene	2,673,584	2,693,584	Calcium-dependent antibiotic	NRPS	5%
Region 16	T2PKS	2,711,900	2,783,355	Spore pigment	t2pks	83%
Region 17	ectoine	2,801,259	2,810,728	Showdomycin	other	52%
Region 18	melanin	3,354,806	3,365,330	Istamycin	saccharide	4%
Region 19	siderophore	3,472,780	3,483,385	Desferrioxamine B	other	83%
Region 20	terpene	3,931,030	3,950,571			
Region 21	T1PKS , NRPS	4,694,531	4,750,189	Thuggacin	nrps-t1pks	12%
Region 22	T2PKS	4,803,448	4,873,883	Oviedomycin	t2pks	95%
Region 23	NRPS	5,093,311	5,144,937	Ikarugamycin	nrps-t1pks	12%
Region 24	NRPS	5,593,128	5,666,806	Marinopyrrole	nrps-t1pks	16%
Region 25	terpene	5,802,574	5,823,000	Albaflavenone	terpene	100%
Region 26	bacteriocin	5,999,330	6,007,931			
Region 27	siderophore	6,427,302	6,439,209	Ficellomycin	NRPS	3%
Region 28	NRPS	6,592,191	6,634,621	Meilingmycin	t1pks	6%
Region 29	bacteriocin	6,694,119	6,703,498	Streptonigrin	other	5%
Region 30	butyrolactone	6,710,173	6,720,639	Rabelomycin	t2pks-saccharide	6%
Region 31	terpene	6,730,307	6,749,776	Geosmin	terpene	100%
Region 32	siderophore	6,889,179	6,902,589			
Region 33	butyrolactone	6,928,260	6,936,962	Griseoviridin / viridogrisein	nrps-transatpks	5%
Region 34	T1PKS	6,938,971	7,013,546	Erythromycin	t1pks	38%
Region 35	butyrolactone , arylpolyene	7,066,089	7,108,766	Rabelomycin	t2pks-saccharide	6%
Region 36	terpene	7,348,015	7,372,444	Hopene	terpene	92%
Region 37	T2PKS , NRPS	7,389,180	7,503,550	Erdacin	t2pks	44%
Region 38	bacteriocin	7,567,823	7,578,178			
Region 39	bacteriocin , NRPS , terpene	7,892,210	7,990,946	Informatipeptin	lanthipeptide	42%
Region 40	NRPS-like	8,207,244	8,249,798	Livipeptin	NRPS	100%
Region 41	NRPS	8,357,875	8,400,853	Disorazole	nrps-transatpks	14%
Region 42	T3PKS	8,451,398	8,492,519	Balhimycin	NRPS	5%
Region 43	nucleoside	8,524,885	8,545,799			
Region 44	ectoine	8,697,094	8,707,507	Ectoine	other	75%
Region 45	lanthipeptide , NRPS	8,940,409	9,046,443	Paulomycin	other	7%
Region 46	fused	9,126,473	9,150,066			

Figure S4: Chromosomal position of the biosynthetic gene clusters predicted by antiSMASH along the chromosome of *Streptomyces sp.* DSM 40868 and *Streptomyces antibioticus* DSM 41481. Metabolites synthetized by similar biosynthetic pathways are signaled by a vertical bar and named.

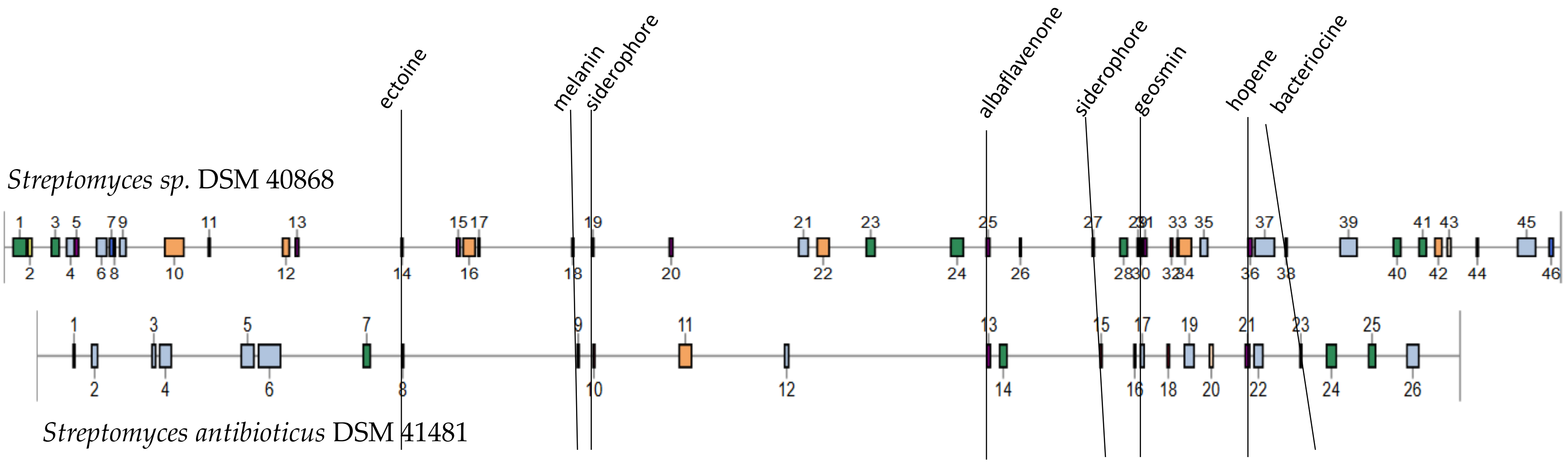

Figure S5 : Comparative analysis of glycolysis/gluconeogenesis pathway of DSM 40868 and DSM 41481.

Streptomyces sp. DSM 40868 | *Streptomyces antibioticus* DSM 41481

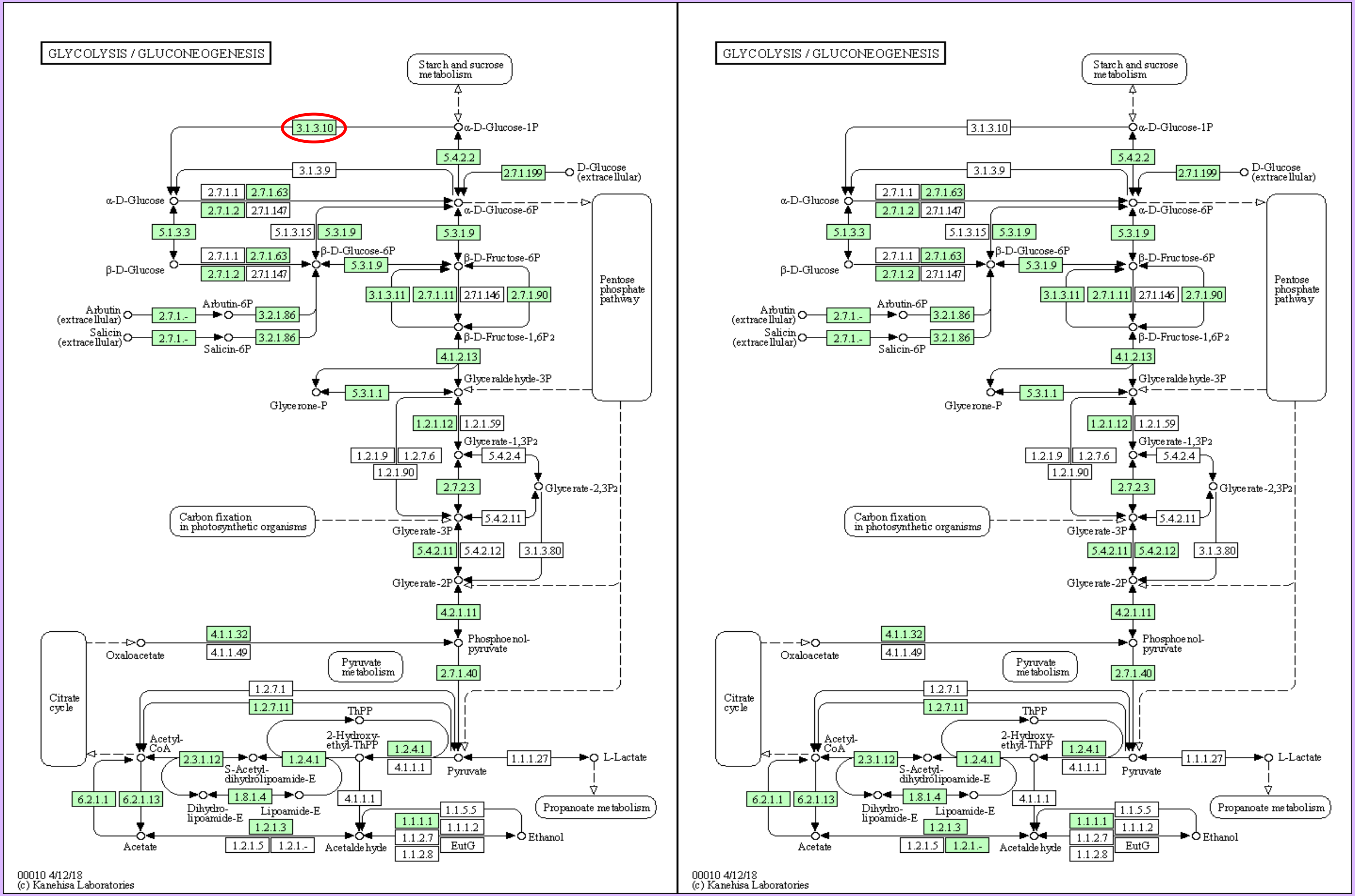

Figure S6 : Comparative analysis of butanoate metabolism of DSM 40868 and DSM 41481.

Streptomyces sp. DSM 40868

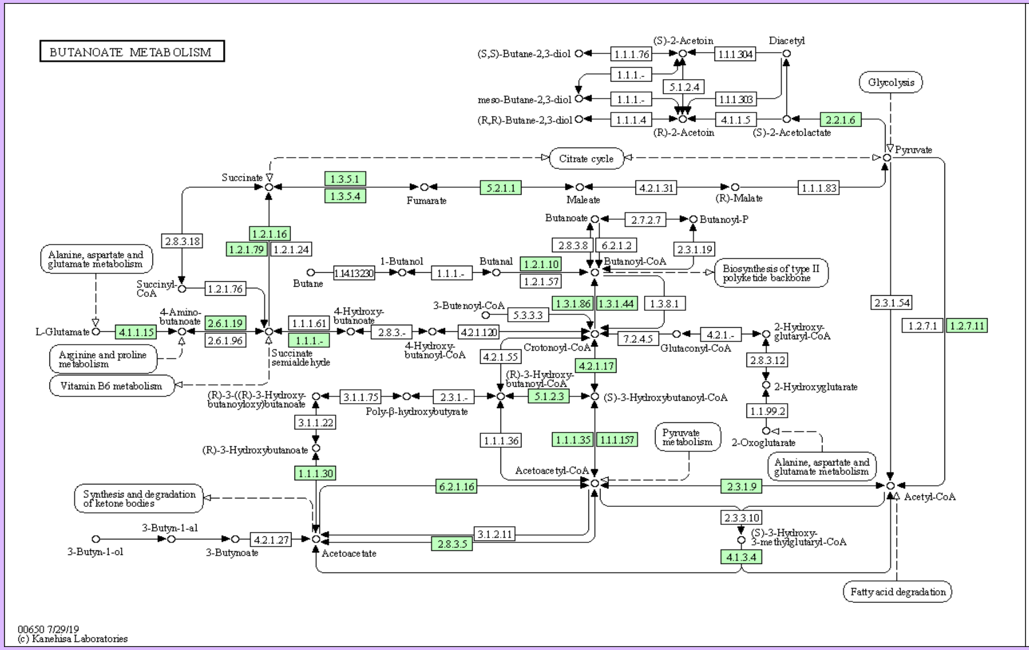

Streptomyces antibioticus DSM 41481

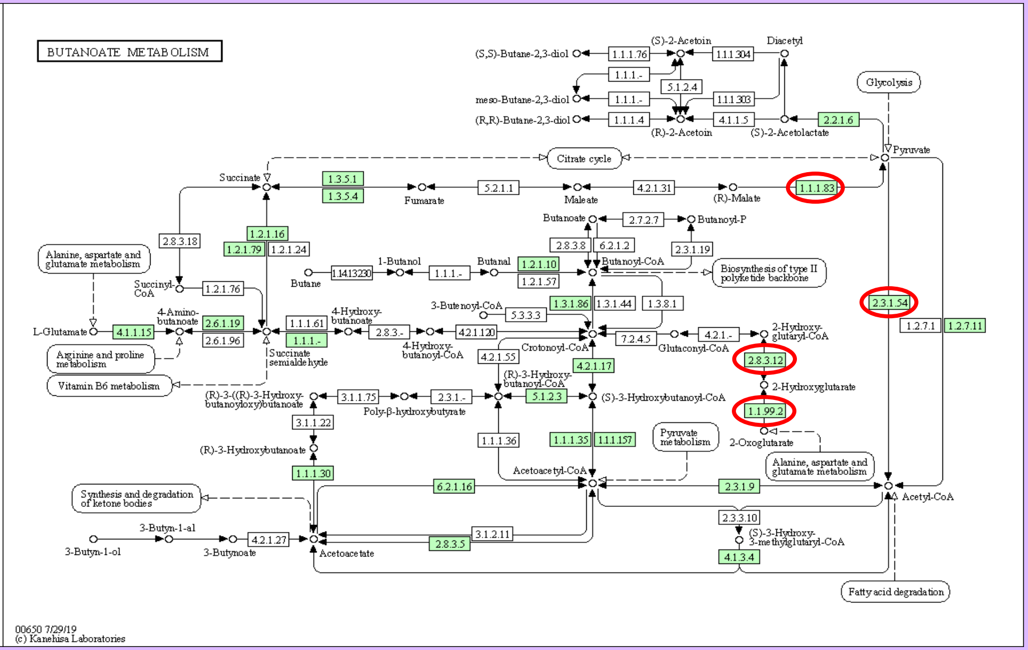

Figure S7 : Comparative analysis of pyruvate metabolism of DSM 40868 and DSM 41481.

Streptomyces sp. DSM 40868 | *Streptomyces antibioticus* DSM 41481

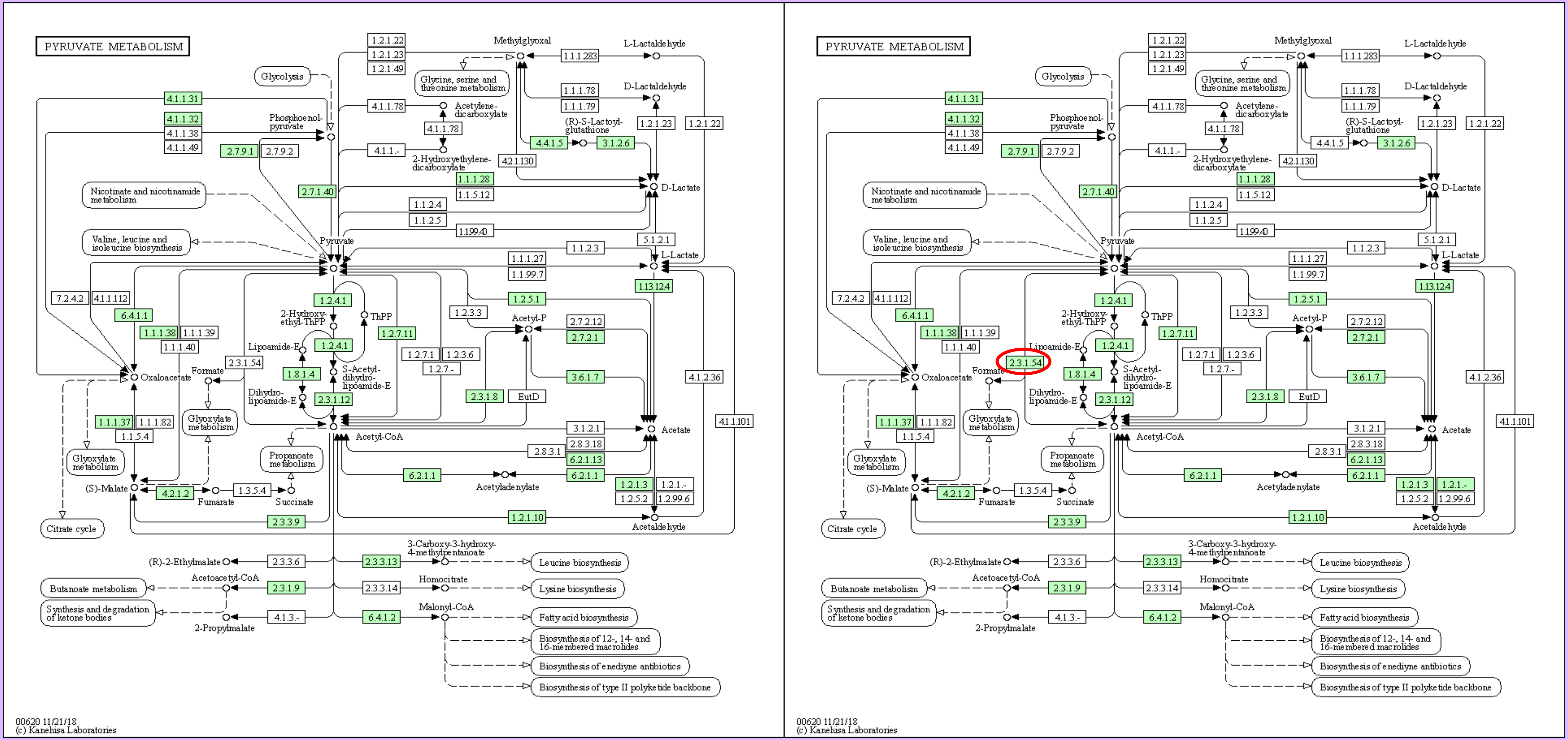

Figure S8 : Comparative analysis of nitrogen metabolism of DSM 40868 and DSM 41481.

Streptomyces sp. DSM 40868

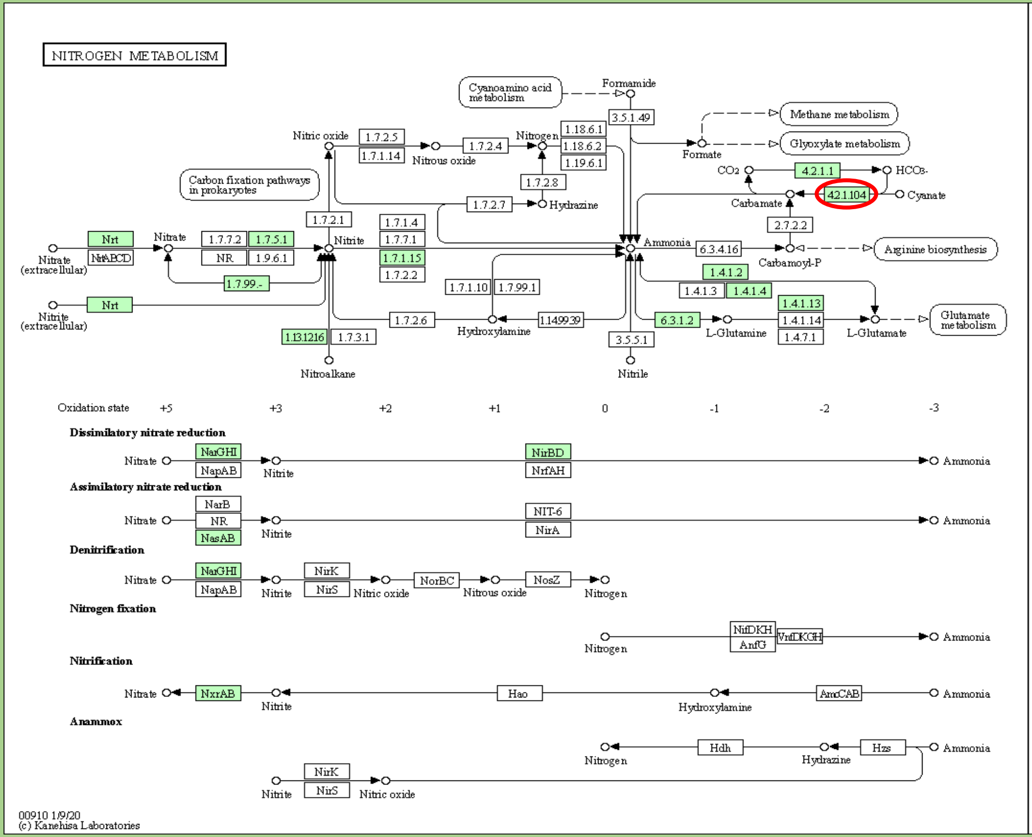

Streptomyces antibioticus DSM 41481

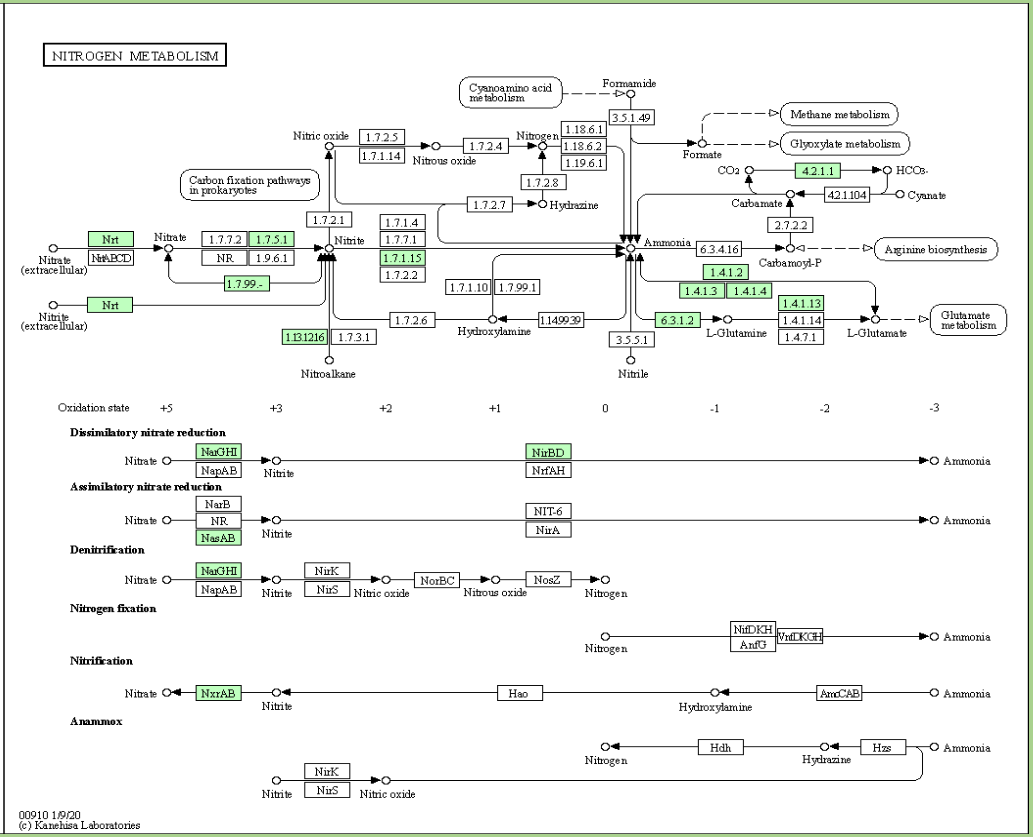

Figure S9: Comparative analysis of glutamine/glutamate metabolism of DSM 40868 and DSM 41481.

Streptomyces sp. DSM 40868 | *Streptomyces antibioticus* DSM 41481

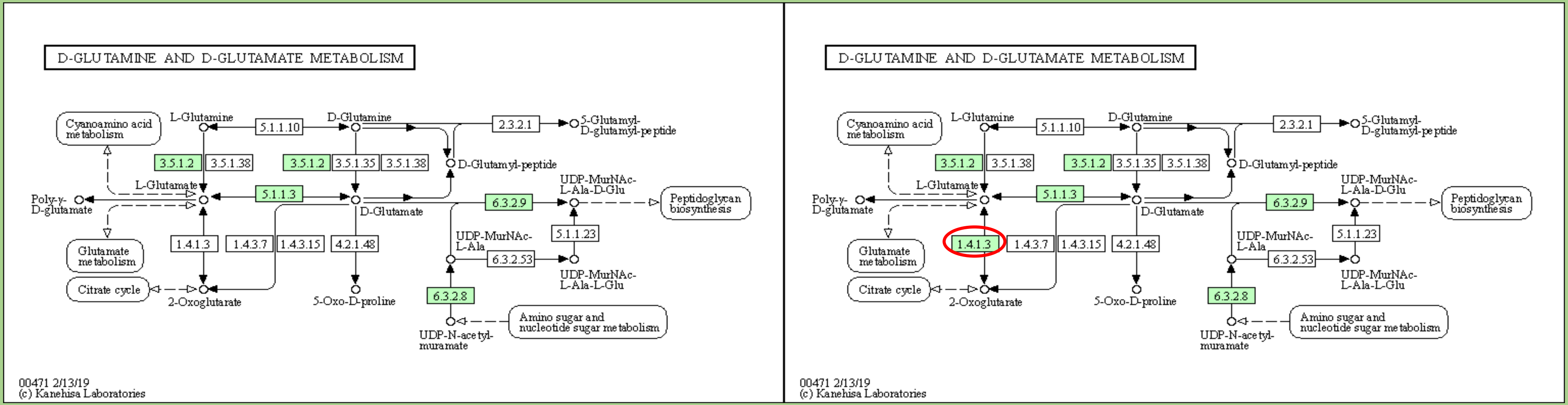

Figure S10 : Comparative analysis of pantothenate and CoA biosynthetic pathways of DSM 40868 and DSM 41481.

Streptomyces sp. DSM 40868

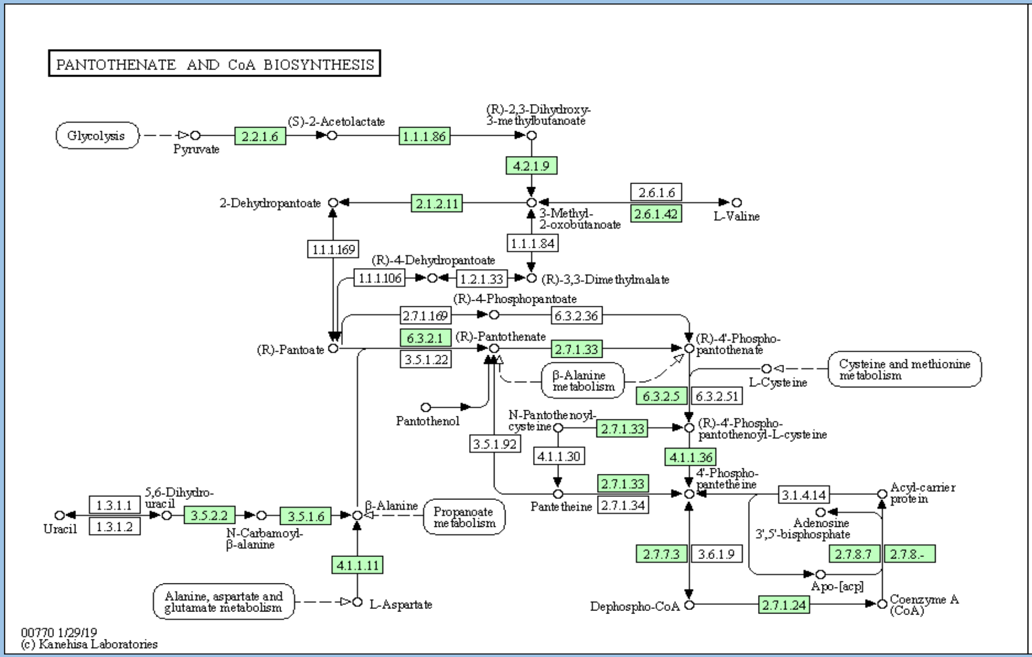

Streptomyces antibioticus DSM 41481

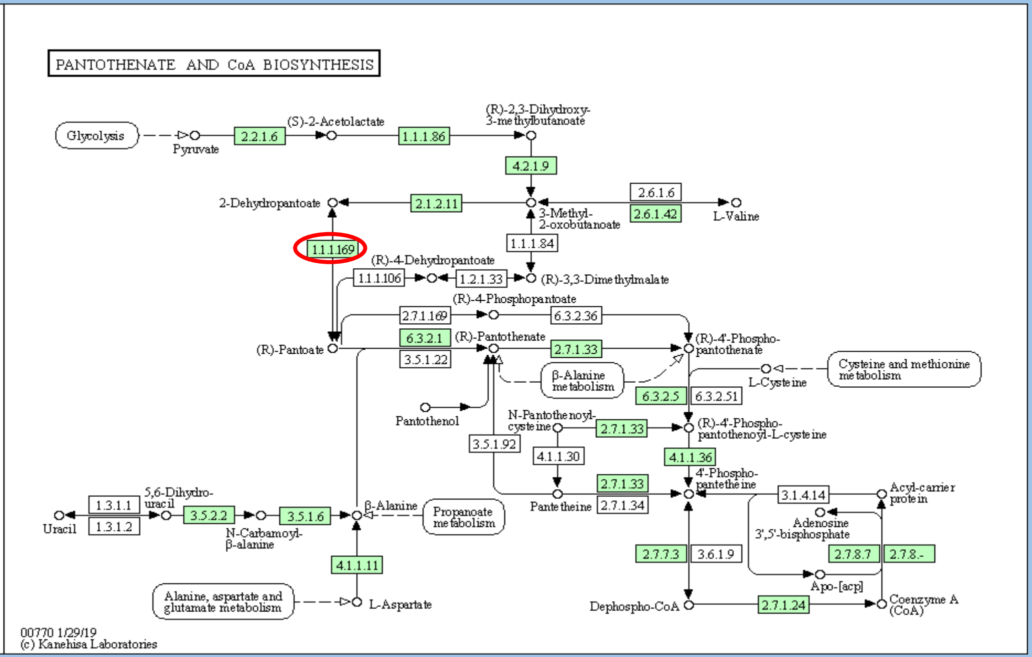

Supplement: Supplementary file 1 [file antibiotics-09-00280-s001.zip › Supplementary figures.pdf]
