# Supplementary material for: Negative Correlation between Lipid Content and Antibiotic Activity in Streptomyces: General Rule and Exceptions
Source: Antibiotics (Basel). 2020 May 26;9(6):280. doi: 10.3390/antibiotics9060280 (PMC7344866; doi:10.3390/antibiotics9060280)
Supplement: Supplementary file 1 [file antibiotics-09-00280-s001.zip › Supplementary Table 1.pdf]

Table S1: Quaternary gradient mobile phase composition. A, isooctane:ethyl acetate (99.8:0.2, v/v); B, acetone:ethyl acetate (2:1, v/v) containing 0.15% acetic acid (v/v); C, 2-propanol:water (85:15, v/v) containing 0.043% acetic acid (v/v) and 0.104% triethylamine (v/v); D, ethyl acetate.

Time (min)	Percent solvent				Flow-rate (mL/min)
	A	B	C	D	
0	100	0	0	0	0.8
1.5	100	0	0	0	0.8
1.6	97	3	0	0	0.8
9	94	6	0	0	0.8
11	70	30	0	0	0.8
14	45	55	0	0	0.8
15	45	55	0	0	0.8
16	40	55	5	0	0.8
20	35	55	10	0	0.8
20.1	33	50	17	0	0.8
25	38	45	17	0	0.8
25.1	48	35	17	0	0.8
30	53	30	17	0	0.8
40	40	0	60	0	0.8
40.1	0	100	0	0	0.8
42	0	100	0	0	0.8
42.1	50	0	0	50	0.8
45	50	0	0	50	0.8
47	100	0	0	0	0.8
53	100	0	0	0	0.8
